# Follow or not? Descriptive norms and public health compliance: Mediating role of risk perception and moderating effect of behavioral visibility

**DOI:** 10.3389/fpsyg.2022.1040218

**Published:** 2022-11-18

**Authors:** Shuwei Zhang, Yan Wang, Yujie Wei

**Affiliations:** ^1^Center for Chinese Public Administration Research, Sun Yat-sen University, Guangzhou, China; ^2^School of Government, Sun Yat-sen University, Guangzhou, China; ^3^College of Economics and Management, South China Agricultural University, Guangzhou, China

**Keywords:** public health compliance behavior, positive norm, negative norm, reference group, risk perception, behavioral visibility

## Abstract

In a pandemic context, public health events are receiving unprecedented attention, and identifying ways to enhance individual public health compliance behaviors has become an urgent practical problem. Considering that individual decisions are susceptible to group members’ behaviors and that descriptive norms provide social information about the typical behaviors of others, we focused on the effects of the properties and reference groups of descriptive norms on public health compliance behaviors. We also investigated the mechanism with risk perception as a mediator and the applicable condition with behavioral visibility as a moderator. Through a 2 × 2 × 2 between-subject survey experiment with 529 subjects, we demonstrated that (1) compared with the negative norm, the positive norm was more effective in promoting public health compliance behaviors; (2) compared with the distal group norm, the proximal group norm more significantly promoted public health compliance behaviors; (3) the effect of the property of descriptive norms on public health compliance behaviors was weakened in the treatment of the proximal group norm; (4) risk perception partially mediated the association between the property of descriptive norms and public health compliance behaviors and fully mediated the effect of the interaction of the property and the reference group of descriptive norms on public health compliance behaviors; in the treatment of the negative-proximal group norm, individuals perceived more risk, thus effectively nudging their public health compliance behaviors; (5) compared with low-visibility behaviors, public health compliance behaviors were significantly stronger for high-visibility behaviors; (6) the property of descriptive norms had a weaker effect on public health compliance behaviors for low-visibility behaviors. In terms of theoretical significance, we refined the study of descriptive norms to promote the application of behavioral public policy. Moreover, the new model of public health compliance behaviors constructed in this study explains the mechanism and applicable conditions of public health compliance behaviors. In practical terms, this study has implications for designing intervention programs to nudge public health compliance behaviors.

## Introduction

In the pandemic context, public health events have received unprecedented attention. The repeated impact of epidemics and the occasional viral outbreaks have increased the uncertainty around people’s life and health, and ways to deal with this uncertainty have become an urgent issue (Wu et al., 2021). The government has formulated a series of scientific and precise public health policies to prevent and control the spread of the pandemic, which is essential to mitigate the challenge of uncertainty (Qian et al., 2021). However, only when citizens actively comply with these public health behaviors can the results of public health policies be realized, and the spread of diseases effectively interrupted (French, 2011; Soofi et al., 2020). Unfortunately, public health policy implementation efforts are often frustrated and the public usually fails to comply with the prescribed public health behaviors. Therefore, promoting public compliance to improve the effectiveness of policy implementation remains a practical challenge (Ang et al., 2021).

Traditional policy intervention tools are based on the “rational man” cognitive model and guaranteed by obligations, mainly in the form of sanctions, prohibitions, and material incentives, but these measures are more costly and less acceptable (Lv et al., 2018) and may also crowd out the intrinsic motivation of individuals to comply with policies (Li et al., 2014). According to the behavioral public policy perspective, individuals’ noncompliance may not result from resistance to policies and may result instead from their bounded rationality and cognitive biases (Lv et al., 2018; Soofi et al., 2020). Therefore, the nudge strategy based on individual decision psychology and behavioral preferences may offer a complementary way to effectively promote individuals’ compliance. In uncertainty situations, individuals prefer to conform to the group members’ behavior (Schultz et al., 2007; Belle and Cantarelli, 2021), and descriptive norms provide individuals with social information about the typical behaviors of others (Cialdini et al., 1991). Previous studies have focused on the role of descriptive norms in individual behaviors, but there is no consensus on which property of descriptive norms is more effective (Hassell and Wyler, 2018; Belle and Cantarelli, 2021), and few studies have made a specific and precise distinction between reference groups of normative information (Zhuang, 2022). Therefore, this study classified descriptive norms in terms of properties and reference groups, focusing on the effect of descriptive norms on public health compliance behaviors.

Additionally, the studies on the mechanism and applicable conditions for descriptive norms in public compliance are still insufficient. Risk perception refers to individuals’ perceived susceptibility to an external threat, which may be a potential mechanism of social factors affecting individual behaviors in uncertain situations (Sitkin and Pablo, 1992). Moreover, behavioral visibility involves the possibility of the behavior being observed by others and may be an indispensable condition for the functioning of descriptive norms (Wismans et al., 2020). Thus, we also considered risk perception and behavioral visibility to further analyze the mechanisms and applicable conditions of descriptive norms.

## Theoretical background and hypothesis formulation

### Public health compliance behaviors

Policy compliance stands for cases in which citizens, as the target group of a policy, follow the public policy measures and undertake behaviors in a manner consistent with the desired policy objectives (Cialdini and Goldstein, 2004). Policy compliance research originated in the 1970s, with early studies focusing on tax policy and exploring ways to promote tax compliance among taxpayers (Allingham and Sandmo, 1972; Fishburn, 1979). As studies emerged, policy compliance research expanded to a variety of fields such as information security (Sommestad et al., 2017), environmental protection (Oliveira Fiorini et al., 2020), health care (Anderson et al., 2020), and crisis management (Muller and Rau, 2021). Researchers have pointed out that policy compliance, as a formal manner of interaction between government and citizens, is an important condition affecting the smoothness of policy implementation and the achievement of policy effects (Liu et al., 2022). Policy compliance in the field of public health is also a major focus of researchers and administrators, as individuals’ active compliance with public health policies is key to controlling the spread of diseases (French, 2011). In a pandemic context, public health compliance behaviors refer not only to daily behaviors such as eating healthy and regularly exercising but also to a series of measures to prevent and control diseases, including washing hands, wearing masks, and maintaining physical distance (Wismans et al., 2020). These policy measures for disease prevention and control are the public health behaviors that we focused on in this study.

Traditional policy compliance studies are based on the “rational man” cognitive model and focus on the impact of the objective policy environment on individual or organizational compliance (Li et al., 2021), involving contributing factors such as powerful authorities (Kastlunger et al., 2013), trust in government (Vu, 2021), and procedural justice (Nagin and Telep, 2017). With the development of behavioral public policy research, the analysis of behavioral characteristics based on the “behavioral man” cognitive model has gradually emerged, focusing on how individual bounded rationality and cognitive biases affect compliance behaviors (Belle and Cantarelli, 2021). Social attributes are one of the individual behavioral characteristics and individuals’ decisions and implementation of behaviors are influenced by behavioral information from social group members (Li et al., 2021); some previous studies have analyzed the association between social norms and individual compliance and confirmed that others’ typical behaviors could influence individual compliance behaviors (Peterson et al., 2021; Rudert and Janke, 2021; Ryoo and Kim, 2021). In this study, we define public health compliance behaviors as the individual’s proactive adoption of disease prevention and control behaviors in a manner expected by public policy goals, focusing primarily on the effect of social norms on public health compliance behaviors and further discussing their mechanism and applicable conditions.

### Descriptive norms and public health compliance behaviors

The term “norm” usually has two meanings: it can either refer to the prevalence or the approval of a behavior. Accordingly, Cialdini et al. (1990) have proposed two types of social norms: one is the individual perception of what most people do, called descriptive norms; the other is the individual perception of what most people morally approve or disapprove of, called injunctive norms. For public health compliance behaviors, individuals overwhelmingly consider these behaviors to be desirable and accepted, so the injunctive norms are stable, clear, and consistent. However, there are significant differences in the individual perception of the actual implementation of public health compliance behaviors. Thus, descriptive norms of public health compliance behaviors are feasible and valuable for research (Guo and Zhang, 2022). Additionally, previous studies have confirmed the more effective role of descriptive norms in influencing individual behavior compared to injunctive norms (Bicchieri and Xiao, 2009; Mollen et al., 2013; Lac and Donaldson, 2018). Therefore, we focused on descriptive norms and explored ways in which individual perceptions of the majority’s behaviors affected individuals’ public health compliance behaviors.

Descriptive norms provide individuals with information on which behaviors are typical, prevalent, and easy to expect in a given situation, which can usually be classified as positive or negative in property: positive norms describe the majority’s behavior and negative norms refer to the majority’s non-behavior (Hassell and Wyler, 2018; Bergquist and Nilsson, 2019). According to the focus theory of normative conduct, individuals tend to automatically look for the majority’s behaviors to guide their own, making the descriptive norms easily become the focus of attention (Nolan et al., 2008). Information on descriptive norms provides individuals with advantages of information processing and shortcuts to decision making, and individuals are likely to simply imitate or conform to what most others are doing when choosing ways to behave in a particular situation (Cialdini et al., 1991; Thaler and Sunstein, 2008). Therefore, under the influence of positive-norm information, individuals tend to follow the majority and perform behaviors actively, while under negative norms, individuals may conform to the majority’s non-behavior and have weak behaviors. For instance, a field experiment on food choices indicated that compared to unhealthy descriptive norms (i.e., negative norms), healthy descriptive norms (i.e., positive norms) led to healthier food choices (Mollen et al., 2013). In the context of COVID-19, some previous studies also discussed the impact of descriptive norms on public health compliance behaviors. Belle and Cantarelli (2021) conducted a series of online survey experiments and found that positive norms were more effective in nudging the vaccination behavior among public employees, which researchers attributed to the conformance effect. Other studies targeting citizens also suggested that positive norms can effectively promote citizens’ current vaccination intentions (Ryoo and Kim, 2021), social distancing (Ang et al., 2021; Rudert and Janke, 2021), and future prevention behaviors (Peterson et al., 2021). Based on the above, we proposed a hypothesis about the properties of descriptive norms as follows:

*H1*: Compared with negative norms, positive norms would be more effective in promoting public health compliance behaviors.

The study of descriptive norms should focus not only on their different properties but also on the different reference groups of the norms. The reference group of descriptive norms is a defined social group used as a standard or an anchor for behavioral reference and comparison (Borsari and Carey, 2003; Mertens and Schultz, 2021). Many previous studies on descriptive norms did not distinguish the reference groups precisely but referred to everyone collectively (Bai and Bai, 2020), or specifically to residents in the neighborhood (Schultz et al., 2014), peers (Rimal, 2008), colleagues (Belle and Cantarelli, 2021), or parents and friends (Lehto et al., 2016). Little is known about which reference group exerts a stronger normative influence on individual behaviors (Zhuang, 2022). However, a critical condition for the proper functioning of descriptive norms is to precisely locate the reference group (Mertens and Schultz, 2021). Different reference groups may have a different impact on individual behaviors, which could be influenced by closeness, proximity, and importance (Leal et al., 2014). Therefore, specifying the reference group facilitates a more effective function of descriptive norms. Considering that, in the context of public health policy, society and the government pay much attention to the physical and spatial distance between people (Qian et al., 2021), we divided the reference groups of descriptive norms into proximal and distal groups according to the actual physical distance between individuals and reference group members. The proximal group norms refer to family members, friends, colleagues, and neighbors who are physically closer to the individual, whereas the distal group norms involve general citizens who are physically further away from the individual.

When information on descriptive norms is from a proximal group, individuals’ attention may be unconsciously increased because it is more relevant to them. According to the focus theory of normative conduct, normative information that attracts individuals’ attention may be more effective in influencing individual behaviors (Cialdini et al., 1991). A recent study on COVID-19 vaccination promotion has confirmed that social norms existing in a proximity reference group were indeed more influential in shaping individual vaccination behavior than social norms in a distal group (Zhuang, 2022). Coincidentally, another study also found that descriptive norms from friends (i.e., a proximal group) could positively predict college students’ exercise and healthy diet behaviors, while descriptive norms from students in the university (i.e., a distal group) did not significantly relate to these behaviors (Yun and Silk, 2011). In the topic of alcoholism, which often involves the reference group of descriptive norms, scholars have argued that norms in a proximal group were more effective in predicting individual alcohol misuse (Voogt et al., 2012; Hagler et al., 2017). Therefore, we proposed our hypothesis about the reference groups of descriptive norms:

*H2*: Compared with distal group norms, proximal group norms would be more significant in promoting public health compliance behaviors*.*

The properties and reference groups of descriptive norms may interact to influence individual compliance behaviors. Researchers have pointed out that the effect of descriptive norms on individual behaviors was dependent on the reference group (Ecker et al., 2019; Alhabash et al., 2021). In our study context, as public health compliance behaviors are closely related to individual life safety, individuals may have strong survival concerns when they perceive noncompliance from the majority of people around them (Wise et al., 2020). Thus, in the condition of a proximal group, negative norms may play an effective role, so that the difference in the effect of positive and negative norms may be weakened. We propose a hypothesis for their interaction as follows:

*H3*: Proximal group norms would weaken the effect of the properties of descriptive norms on public health compliance behaviors*.*

### The mediating role of risk perception

Risk perception refers to an individual’s perceived susceptibility to a threat, which can significantly influence individual risk response behaviors (Ferrer and Klein, 2015). Rogers (1975) introduced the protection motivation theory to explain how protective behaviors were initiated or maintained in the presence of a threat stimulus. According to the model of behavior formation, the protection motivation theory includes three components: sources of information, cognitive mediating processes, and coping modes, while the cognitive mediating process involves threat appraisal and coping appraisal (Rogers, 1983). Threat information from environmental and interpersonal sources triggers individual risk perceptions, motivating individuals to assess the level of threat and their response-ability for mitigating threats and leading to subsequent protective behaviors (Floyd et al., 2000; Zhu et al., 2022). In the context of COVID-19, protection motivation theory has been widely used in the public health field to explain the effect of risk perception on individual preventive behaviors (Kowalski and Black, 2021; Lahiri et al., 2021; Tu et al., 2022).

Risk perception is an important factor in predicting individual behaviors in risky situations, and social factors can indirectly affect individual protective behaviors through the mediating role of risk perception (Sitkin and Pablo, 1992). Previous studies have confirmed the high correlation between descriptive norms and risk perception (Carter et al., 2014), and that the prevalence of health-hazardous behaviors increases individual perceived risk and thus promotes health-related behaviors (Li et al., 2017). In the public health context, when individuals observe that most people do not comply with public health policies, their perceived risk of the social environment and perceived possibility of self-exposure to risk are increased, and according to protection motivation theory, the perception of high risk at this time promotes individuals’ public health compliance behaviors to protect themselves (Idrees et al., 2022). In addition, individuals’ assessment of risk threats is often influenced by the physical distance between the risk and themselves (Planas et al., 2021; Di Guilmi et al., 2022). Therefore, information on descriptive norms from proximal groups such as neighbors, family, friends, and colleagues may have a stronger impact on individual risk perceptions (Barnum and Armstrong, 2019), and thus more effectively promote individual behaviors. Moreover, the proximal group may amplify the role of normative information on individual risk perception (Wise et al., 2020). When individuals perceive that most people around them are not complying with public health policies, they may reach a high level of susceptibility and severity and thus be more willing to adopt a self-protective coping mode and actively practice public health behaviors. Therefore, we hypothesized that risk perception would mediate the association between descriptive norms and public health compliance behaviors. Specifically, we proposed the following three hypotheses:

*H4-1*: Risk perception would mediate the association between the properties of descriptive norms and public health compliance behaviors*.*

*H4-2*: Risk perception would mediate the association between the reference groups of descriptive norms and public health compliance behaviors*.*

*H4-3*: Risk perception would mediate the association between the interaction of the properties and reference groups of descriptive norms and public health compliance behaviors*.*

### The moderating effect of behavioral visibility

Public health policy involves different types of public health measures. In the context of COVID-19, the government has proposed more public health behaviors that individuals should comply with. Public health behaviors cannot simply be understood as a sole behavioral construct, because different characteristics of behaviors may have different effects on individual compliance (Wismans et al., 2020). Behavioral visibility is an important characteristic of public health behaviors, and it is defined as the performance of the individual behavior that can be observed by others through minimal effort (Leonardi and Treem, 2020). Behavioral visibility has been shown to affect individual compliance behaviors in the field of information security and voter mobilization (Panagopoulos, 2011; Hwang et al., 2017). When public health behaviors can be easily observed by others, individuals may be more motivated to comply with them, while when they are less visible, individuals may become unconcerned and reduce their public health behaviors. Therefore, we proposed the hypothesis of behavioral visibility as follows:

*H5*: Individuals would have higher compliance with high-visibility public health behaviors compared to low-visibility public health behaviors*.*

Descriptive norms promote individual compliance behaviors, in part because individuals observe the prevalence of the behavior and create social pressure to conform to the majority (Blais et al., 2018). When individuals perceive that the prevalence and visibility of the behavior is high, their conformity pressure may be increased, resulting in stronger compliance behaviors (Wismans et al., 2020). Conversely, when compliance behaviors are not easily observed by others, the facilitative effect of normative information on individual compliance behaviors may be diminished by the low visibility of behaviors. Additionally, behavioral visibility may also moderate the effect of the reference group of descriptive norms on public health compliance behaviors. For high-visibility behaviors, individuals’ perception that the proximal group is performing the behavior may promote them to actively display behaviors consistent with those around them (Belle and Cantarelli, 2021). For low-visibility behaviors, individuals may think that the consistency of their behaviors with those around them cannot be observed, thus the information on proximal group norms may not effectively improve individual compliance behaviors. Based on the above, we hypothesized the moderating role of behavioral visibility in the association between descriptive norms and public health compliance behaviors. The specific hypotheses are as follows:

*H6-1*: Behavioral visibility would moderate the effect of the properties of descriptive norms on public health compliance behaviors. Specifically, the effect of the properties of descriptive norms on public health compliance behaviors would be stronger for high-visibility behaviors than for low-visibility behaviors*.*

*H6-2*: Behavioral visibility would moderate the effect of the reference groups of descriptive norms on public health compliance behaviors. Specifically, the effect of the reference groups of descriptive norms on public health compliance behaviors would be stronger for high-visibility behaviors than for low-visibility behaviors*.*

**Figure 1:**
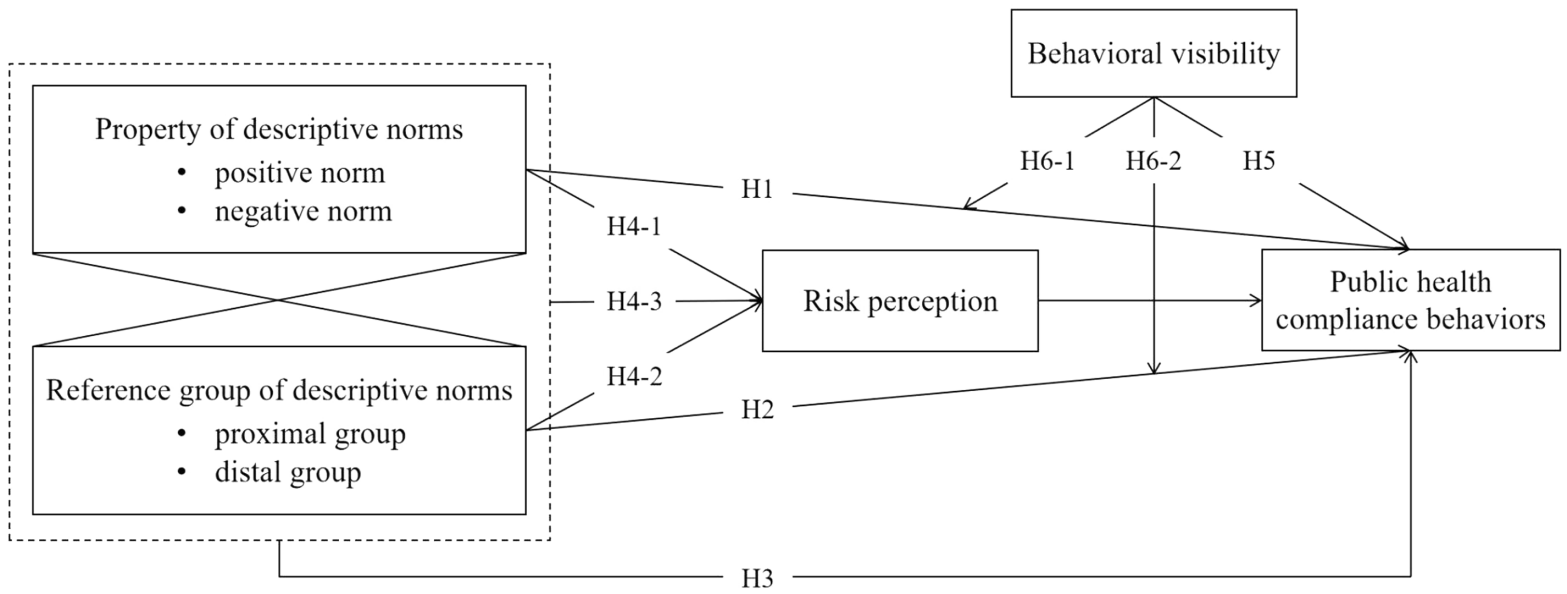
The hypothesized model.Note. The dotted box represents the interaction between the property and the reference group of descriptive norms.

All hypotheses were presented in Figure 1.

## Methods

### Experiment design

We designed a 2 (property: positive norm vs. negative norm) × 2 (reference group: proximal group vs. distal group) × 2 (behavioral visibility: high visibility vs. low visibility) between-subjects survey experiment, in which subjects were randomly assigned to one of eight experimental conditions. In the experimental questionnaire, we created a virtual policy situation of “The spring influenza virus broke out in City A, and its government urged citizens to strengthen their personal hygiene and health management,” and artificially designed news reports and community notifications to improve the realistic compatibility of experimental materials and facilitate subjects’ smooth entry into the experimental situation. The method of manipulating experimental variables in a virtual situation has been widely used in previous survey experiments (Martini and Olmastroni, 2021). This study obtained ethical approval from Institutional Review Board Office, School of Government, Sun Yat-sen University.

### Procedure

**Figure 2:**
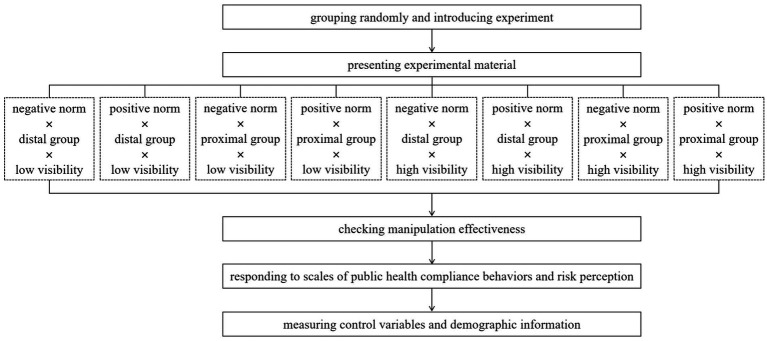
The procedure of the survey experiment.

Upon entering the experiment, subjects were randomly assigned to one of eight experimental groups. First, they were presented with an introductory paragraph and pictures to guide them into the policy situation of this experiment. Then, they were exposed to the experimental material that corresponds to their experimental condition. After reading the text, subjects were required to respond to the manipulation check items of the properties and reference groups of descriptive norms and behavioral visibility. Next, subjects were instructed to answer questions from scales of public health compliance behaviors and risk perception. Finally, the subjects’ risk-seeking and agreeableness were assessed and they provided their demographic information. A plot of the experimental procedure is shown in Figure 2.

### Participants

We conducted the online survey experiment through the Credamo data platform. The Credamo data platform is a professional data collection platform in China with 2.8 million participants, providing research data services to over 2,000 universities worldwide. Its sample quality has been recognized by many international journals in the field of psychology, including *Psychological Science*, *Journal of Personality and Social Psychology*, and *Frontiers in Psychology* (Gong et al., 2020; Jiang and Sedikides, 2021; Xiao et al., 2021).

We used G*Power software version 3.1.9.2 to prior estimate the sample size before the experiment. The statistical power level was set to 95%, the significance level was set to 0.05, and the effect size was set to 0.25 (Faul et al., 2007). The total required sample size was calculated to be at least 210 subjects, with no less than 27 subjects per group. A total of 699 questionnaires were administered and all were returned. Then, we screened them according to the following inclusion criteria: (1) the response duration was no less than 110 s, which was the shortest duration that the researcher has obtained from repeated simulations of the experiment; (2) passing the screening questions set by the researcher in advance; (3) no obvious problems, such as highly consistent or contradictory answers to different items. Finally, 529 valid questionnaires remained (*N_negative × distal×low_* = 70, *N_positive × distal×low_* = 68, *N_negative × proximal×low_* = 68, *N_positive × proximal×low_* = 68, *N_negative × distal×high_* = 60, *N_positive × distal×high_* = 70, *N_negative × proximal×high_* = 60, *N_positive × proximal×high_* = 65), which met the sample size requirement obtained by the prior estimation. Table 1 presented the subjects’ demographic information. Additionally, we conducted inter-group difference tests and found no significant differences in demographic variables under different experimental conditions (*p_all_* > 0.05), which indicated that the random assignment was effective.

**Table 1:** Demographic information of subjects.

	M ± SD (range) / *N* (%)
Age	29.54 ± 8.40 (18–65)
Gender	
Male	202 (38.2%)
Female	327 (61.8%)
Education	
Tertiary education or below	112 (21.2%)
Undergraduate education	356 (67.3%)
Postgraduate education	61 (11.5%)
Political status	
Communist Party of China	109 (20.6%)
Communist Youth League	180 (34.0%)
Other parties or nonparty	240 (45.4%)
Annual income (RMB)	
≤ 60,000	108 (20.4%)
60,001–100,000	147 (27.8%)
100,001–200,000	196 (37.1%)
> 200,000	78 (14.7%)
Type of occupation	
Student	120 (22.7%)
Public servants or public institution	120 (22.7%)
State-owned enterprise	70 (13.2%)
Private enterprise	192 (36.3%)
Others	27 (5.1%)
Marital status	
With a spouse	252 (47.6%)
Single (unmarried/divorced/ widowed)	277 (52.4%)

### Variable manipulation

*The properties of descriptive norms* comprised positive norm and negative norm. The experimental material under the treatment condition of a positive norm identified that most individuals actively complied with public health behaviors advocated by the government. Specifically, the text clearly indicated that over 75% of individuals actively practiced public health norms, presented the interview content of respondents’ active participation in public health behaviors, and repeatedly used the words “most of the time, every time, and great attention” to strengthen the manipulation for the positive norm. Conversely, the experimental material under the treatment condition of a negative norm identified that most individuals did not comply with public health behaviors. The specific text indicated that over 75% of individuals were very inattentive to public health management, presented interviews in which respondents explicitly stated that they did not follow the public health practices promoted by the government, and repeatedly used the words “rarely, basically not, not much attention” to strengthen the manipulation for the negative norm.

*The reference group of descriptive norms* comprised proximal and distal group. In the experimental material referring to the proximal group, subjects read a community notification issued by the community committee, and the participants in the data survey and interview were neighbors, friends, family members, and colleagues who had closer physical proximity to the individuals. In contrast, in the experimental material referring to the distal group, subjects read a news report published in the morning newspaper of City A, and the participants in the survey and interview were general citizens who were more physically distant from the individuals.

*Behavioral visibility* comprised high and low visibility. We chose the behavior of mask-wearing as the high-visibility public health behaviors in this study. In the context of COVID-19, wearing masks has become a common public health behavior. The behavior of wearing masks usually lasts for a long time and is mostly in public spaces, especially in crowded places, so wearing masks is easily observed by others, which meets the requirement for high behavioral visibility in this experiment. For the low-visibility public health behaviors, we selected hand hygiene behaviors, that is, a collective term of hygiene behaviors that reduce the retention of transient flora on the skin surface of the hands. Hand hygiene behaviors are usually performed for a short period of time and in a private place, usually at home or in public restrooms, so they meet the experimental requirement for low behavioral visibility. The complete experimental materials were presented in Appendix 1.

### Variable measurement

#### Manipulation check

Subjects were asked to respond to three items to separately examine whether the interventions of the three manipulated variables were significantly effective in this study. The items were: as a resident of City A: (1) I think [most people in this city]/[most people among my neighbors, friends, family members, and colleagues] follow [hand hygiene behaviors]/[mask wearing behaviors]; (2) I think [the citizens in this city]/[my neighbors, friends, family members, and colleagues] are physically close to me; (3) I think others can notice whether [I am wearing a mask]/[I am doing hand cleaning]. Subjects responded to the items on a seven-point scale, ranging from “1” (strongly disagree) to “7” (strongly agree).

#### Public health compliance behaviors

Based on manipulation of the behavioral visibility, public health compliance behaviors specifically refer to mask-wearing or hand hygiene behaviors in this study. The specific measurement items refer to the measures of hand hygiene and mask wearing in the guidelines for preventive behavior introduced by the World Health Organization and the European Centre for Disease Prevention and Control. Hand hygiene behavior and mask-wearing behavior measurements each contain five items, rated on an 11-point Likert scale, from “1” (strongly disagree) to “11” (strongly agree). For the complete items, please see Appendix 2. We calculated the average score of the five items as the subjects’ scores in public health compliance behaviors, with a higher score representing stronger compliance behaviors. In this study, Cronbach’s α values were 0.87 for hand hygiene behaviors, and 0.90 for mask-wearing behaviors.

#### Risk perception

We adapted the items from the HIV study by Napper et al. (2012) and the SARS study by Brug et al. (2004) to form a risk perception measurement scale in a public health context. The measurement contains six items, with response options of “1” (strongly disagree) to “11” (strongly agree). For the complete items, please see Appendix 2. After reversing the score of the reverse-scoring item, we calculated the average score of the six items as the subjects’ risk perception score. In this study, Cronbach’s α value for risk perception was 0.78.

#### Control variable

Considering that differences in risk-seeking might influence individual behaviors even if they have the same level of risk perception, we included risk-seeking as a control variable in this study. The measurement of risk-seeking contains four items, which were adapted from a traditional gambling task questionnaire introduced by Liu et al. (2010). Participants chose one of two options, with no points for the conservative option and five points for the risky option. We used the mean of subjects’ scores on four items as their risk-seeking score. In addition, if an individual is naturally more agreeable, his compliance behavior might naturally be higher, so agreeableness should be included as a control variable. Items of agreeableness were adapted from the agreeableness dimension in the Chinese Big Five Personality Inventory by Wang et al. (2011), with a seven-point scale from “1” (strongly disagree) to “7” (strongly agree). After reversing the score of reverse-scoring items, we used the average score of five items as the subjects’ score of agreeableness. Demographic variables included gender, age, education, political status, income, type of occupation, and marital status.

## Results

### Manipulation check

The results of the independent sample t-tests revealed a significant difference in the subject’s perceived prevalence of others’ compliance with public health behaviors reflected in the experimental text under the positive or negative norms (*M_positive_* = 6.20, *SD_positive_* = 0.85; *M_negative_* = 2.62, *SD_negative_* = 1.60; *t* = 31.93, *p* < 0.001, *Cohen’s d* = 2.82). The subject’s perception of the physical distance of others shown in the experimental text also differed significantly for the proximal and distal groups (*M_proximal_* = 5.44, *SD_proximal_* = 1.30; *M_distal_* = 3.88, *SD_distal_* = 1.63; *t* = 12.15, *p* < 0.001, *Cohen’s d* = 1.05). In addition, there was also a significant difference in the subjects’ perceived visibility of the behavior involved in the experimental text under high-visibility or low-visibility behaviors (*M_high_* = 5.69, *SD_high_* = 1.32; *M_low_* = 4.02, *SD_low_* = 1.73; *t* = 12.58, *p* < 0.001, *Cohen’s d* = 1.08). Therefore, the effectiveness of the manipulation of the three manipulated variables—the properties of descriptive norms, the reference groups of descriptive norms, and the behavioral visibility—were remarkably effective.

### The main effects of the properties and the reference groups of descriptive norms on public health compliance behaviors

From the ANOVA results, compared with individuals presented with the negative norm, the public health compliance behavior of individuals exposed to positive-norm materials was significantly higher (*M_positive_* = 9.37, *SD_positive_* = 1.18; *M_negative_* = 8.59, *SD_negative_* = 1.39; *F* = 49.72, *p* < 0.001, η^2^ = 0.09; see Table 2). Additionally, compared with individuals exposed to distal group materials, the public health compliance behavior of individuals given proximal-group materials was significantly higher (*M_proximal_* = 9.16, *SD_proximal_* = 1.11; *M_distal_* = 8.82, *SD_distal_* = 1.52; *F* = 10.07, *p* < 0.01, η^2^ = 0.02; see Table 2). Thus, Hypotheses 1 and 2 were supported.

**Table 2:** The main effects and the interaction effect of the property and the reference group of descriptive norms on public health compliance behaviors.

Independent variables		*M* ± *SD*	*F*	*df*	η^2^
Property	Positive norm (*N* = 271)	9.37 ± 1.18	49.72^***^	1	0.09
	Negative norm (*N* = 258)	8.59 ± 1.39			
Reference group	Proximal group (*N* = 261)	9.16 ± 1.11	10.07^**^	1	0.02
	Distal group (*N* = 268)	8.82 ± 1.52			
Property × Reference group			6.31^*^	1	0.01

*M* = Mean, *SD* = Standard deviation.

* *p* < 0.05;

** *p* < 0.01;

*** *p* < 0.001.

### The interaction effect between the properties and reference groups of descriptive norms on public health compliance behaviors

**Figure 3:**
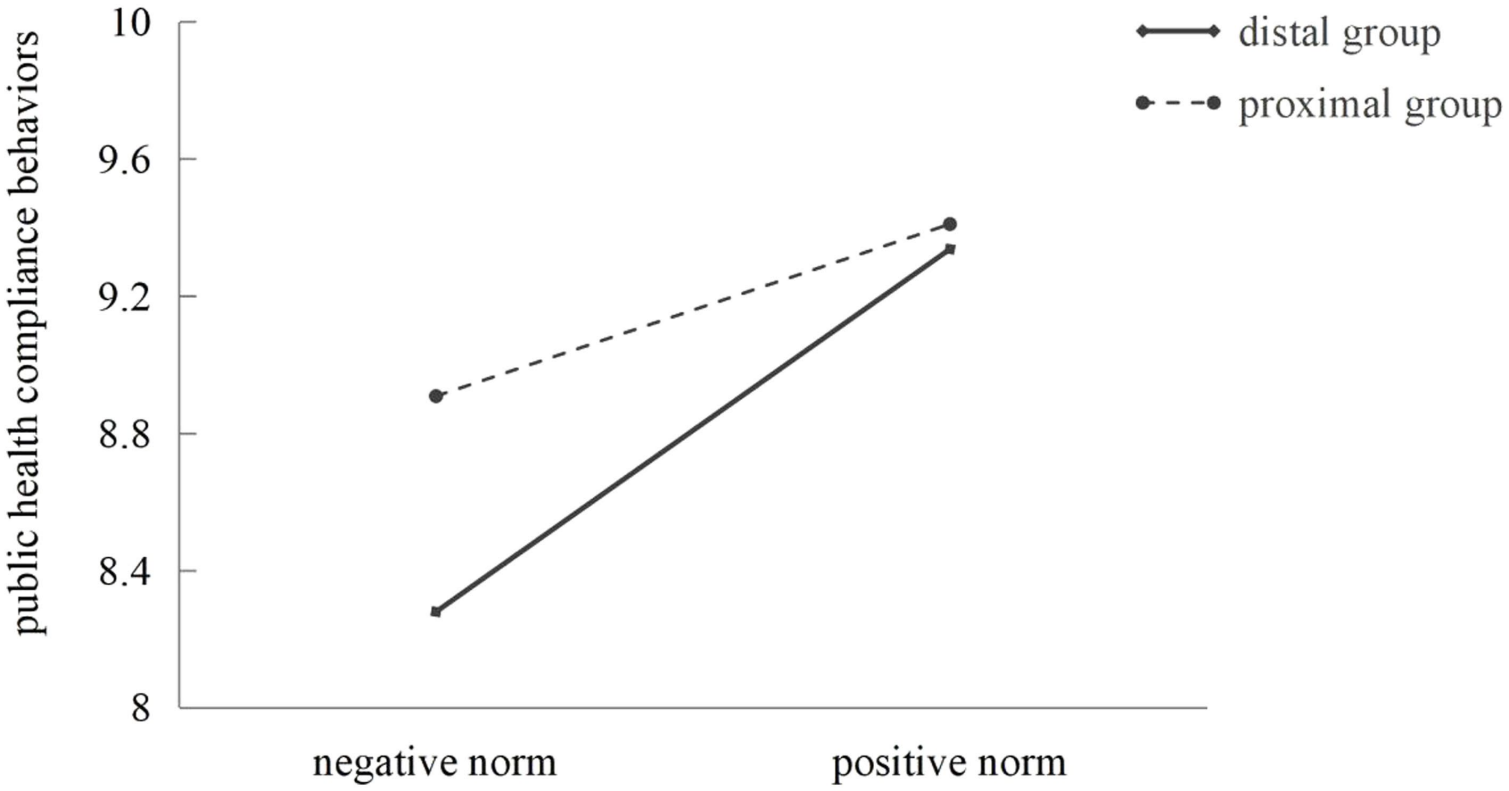
The interaction effect of the property and the reference group of descriptive norms on public health compliance behaviors.

According to the ANOVA results, the interaction between the properties and reference groups of descriptive norms was significant (*F* = 6.31, *p* < 0.05, η^2^ = 0.01; see Table 2). To further interpret this interaction result, we graphed the interaction effect in Figure 3. Compared with the distal group, the effect of the properties of descriptive norms on public health compliance behaviors was weakened in the proximal group scenario. Meanwhile, analyses of simple effects revealed that the difference between the positive and negative norms under the proximal group (*M_positive − negative_* = 0.50, *F* = 10.17, *p* < 0.01) was smaller than that under the distal group (*M_positive − negative_* = 1.06, *F* = 46.32, *p* < 0.001). Based on the above results, Hypothesis 3 was supported.

### The mediating role of risk perception

The indirect effect of the properties of descriptive norms on public health compliance behaviors through risk perception was presented in Table 3. After we included control variables in the regression model, the property of descriptive norms had a negative direct effect on risk perception (*β* = −0.44, *p* < 0.001; see Table 3), risk perception had a positive direct effect on compliance behaviors (*β* = 0.63, *p* < 0.001; see Table 3), and the property of descriptive norms still had a positive direct effect on compliance behaviors (*β* = 0.83, *p* < 0.001; see Table 3). Considering that the value of interactions may not necessarily follow a normal distribution, we conducted a bootstrapping procedure 5,000 times to estimate the indirect effect and constructed confidence intervals (CI) for the indirect effect. The indirect effect of the properties of descriptive norms on compliance behaviors through risk perception was significant (β = −0.28, 95% CI [−0.3892, −0.1794]; see Table 3), which supported Hypothesis 4–1. However, the indirect effect of the reference group of descriptive norms on compliance behaviors through risk perception was not significant (*β* = 0.02, 95% CI [−0.0655, 0.1171]). Thus, Hypothesis 4-2 was not supported in this study.

**Table 3:** The indirect effect of the property of descriptive norms on public health compliance behaviors through risk perception (*N* = 529).

Variable	Risk perception	Public health compliance behaviors
Intercept	0.27	−0.44
Property	−0.44^***^	0.83^***^
Risk perception		0.63^***^
Indirect effects [95% bootstrap CI]		−0.28 [−0.3892, −0.1794]
*R* ^2^	0.11^***^	0.49^***^

CI = Confidence intervals. Bootstrap = 5,000. Control variables (gender, age, education, political status, annual income, type of occupation, marital status, risk-seeking, and agreeableness) were included in this model but not presented in this table.

*** *p* < 0.001.

**Figure 4:**
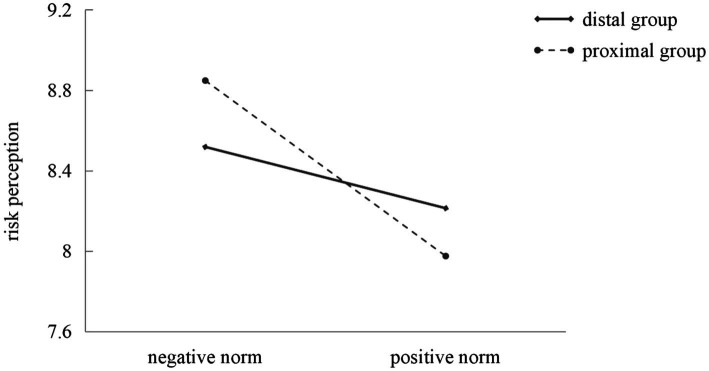
The interaction effect of the property and the reference group of descriptive norms on risk perception.

In addition, Table 4 presented the indirect effect of the interaction between the property and the reference group of descriptive norms on public health compliance behaviors through risk perception. The direct effect of the interaction between the property and the reference group of descriptive norms on risk perception was negatively significant (*β* = −0.40, *p* < 0.05; see Table 4). Specifically, compared with the distal group, the effect of the properties of descriptive norms on risk perception was stronger for the proximal group (see Figure 4). The direct effect of risk perception on compliance behaviors was positively significant (β = 0.62, *p* < 0.001; see Table 4). However, the direct effect of the interaction between the property and the reference group of descriptive norms on compliance behaviors was not significant (β = −0.14, *p* > 0.05; see Table 4). Additionally, the indirect effect of the interaction between the property and the reference group of descriptive norms on public health compliance behaviors through risk perception was significant (*β* = −0.25, 95% CI [−0.4621, −0.0416]; see Table 4). Therefore, Hypothesis 4-3 was confirmed.

**Table 4:** The indirect effect of the interaction between the property and the reference group of descriptive norms on public health compliance behaviors through risk perception (*N* = 529).

Variable	Risk perception	Public health compliance behaviors
Intercept	0.16	−0.59
Property × Reference group	−0.40^*^	−0.14
Risk perception		0.62^***^
Indirect effects [95% bootstrap CI]		−0.25 [−0.4621, −0.0416]
*R* ^2^	0.12^***^	0.50^***^

CI = Confidence intervals. Bootstrap = 5,000. Control variables (gender, age, education, political status, annual income, type of occupation, marital status, risk-seeking, and agreeableness) were included in this model but not presented in this table.

* *p* < 0.05;

*** *p* < 0.001.

### The moderating effect of behavioral visibility

**Figure 5:**
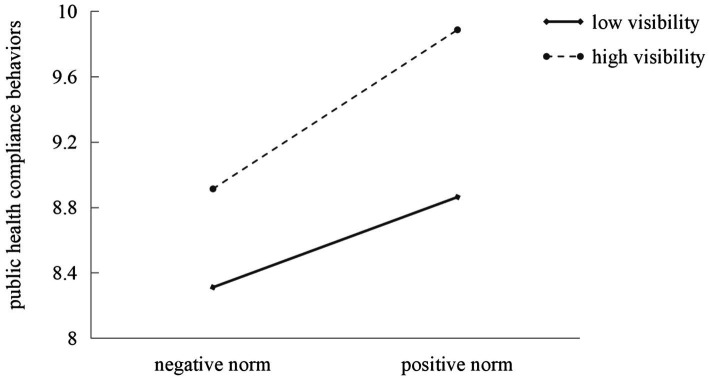
The interaction effect of the property of descriptive norms and the behavioral visibility on public health compliance behaviors.

The ANOVA results presented in Table 5 indicated that public compliance behavior was significantly higher under high behavioral visibility compared to low behavioral visibility (*M_high_* = 9.43, *SD_high_* = 1.36; *M_low_* = 8.59, *SD_low_* = 1.20; *F* = 58.64, *p* < 0.001, η^2^ = 0.10; see Table 5), which supported Hypothesis 5. Moreover, the interaction effect between the property of descriptive norms and behavioral visibility was significant (*F* = 3.93, *p* < 0.05, η^2^ = 0.01; see Table 5). We graphed the interaction results in Figure 5 and found that compared with the behavior with low visibility, the effect of the properties of descriptive norms was significantly stronger for the behavior with high visibility. The results of simple effects also revealed that the difference between the positive and negative norms under high behavioral visibility (*M_positive − negative_* = 0.97, *F* = 40.46, *p* < 0.001) was greater than that under low behavioral visibility (*M_positive − negative_* = 0.55, *F* = 14.05, *p* < 0.001). Therefore, Hypothesis 6–1 was confirmed. However, the interaction effect between the reference groups of descriptive norms and behavioral visibility was not significant (*F* = 0.75, *p* > 0.05), which did not support Hypothesis 6–2. The summary of the study hypothesis testing was presented in Table 6.

**Table 5:** The main effects and the interaction effect of the property of descriptive norms and behavioral visibility on public health compliance behaviors.

Independent variables		*M* ± *SD*	*F*	*df*	η^2^
Property	Positive norm (*N* = 271)	9.37 ± 1.18	51.58^***^	1	0.09
	Negative norm (*N* = 258)	8.59 ± 1.39			
Behavioral visibility	High visibility (*N* = 255)	9.43 ± 1.36	58.64^***^	1	0.10
	Low visibility (*N* = 274)	8.59 ± 1.20			
Property × Behavioral visibility			3.93^*^	1	0.01

*M* = Mean, *SD* = Standard deviation.

* *p* < 0.05;

*** *p* < 0.001.

**Table 6:** Summary of the study hypothesis testing.

Specific hypothesis	Results
H1: Compared with negative norms, positive norms would be more effective in promoting public health compliance behaviors.	Supported
H2: Compared with distal group norms, proximal group norms would be more significant in promoting public health compliance behaviors.	Supported
H3: Proximal group norms would weaken the effect of the properties of descriptive norms on public health compliance behaviors.	Supported
H4-1: Risk perception would mediate the association between the properties of descriptive norms and public health compliance behaviors.	Supported
H4-2: Risk perception would mediate the association between the reference groups of descriptive norms and public health compliance behaviors.	Not supported
H4-3: Risk perception would mediate the association between the interaction of the properties and reference groups of descriptive norms and public health compliance behaviors.	Supported
H5: Individuals would have higher compliance with high-visibility public health behaviors compared to low-visibility public health behaviors.	Supported
H6-1: Behavioral visibility would moderate the effect of the properties of descriptive norms on public health compliance behaviors.	Supported
H6-2: Behavioral visibility would moderate the effect of the reference groups of descriptive norms on public health compliance behaviors.	Not supported

## Discussion

### The main effect of the properties of descriptive norms: Follow the herd

We found that the positive norm was more effective in promoting public health compliance behaviors than the negative norm, which is consistent with the focus theory of normative conduct (Cialdini et al., 1991). Positive norms provide individuals with information about the prevalence of the behavior, and in the face of the advantages of information processing and shortcuts to decision making, individuals prefer to comply with the majority’s behaviors rather than being outliers (Floyd et al., 2000; Thaler and Sunstein, 2008). Thus, when individuals perceive that most people are complying with public health policies, they may tend to join them and actively implement public health compliance behaviors. Although compliance behaviors can be cumbersome to implement, imitating others to comply with public health policies is likely more effective, adaptive, and sensible at this time. In addition, availability heuristics also provides a possible explanatory perspective for the effectiveness of positive norms (Kahneman, 2011). Positive norms can trigger availability heuristics and make the idea of compliance with public health policies more accessible in individuals’ minds, which can unconsciously and rapidly enhance individual compliance behaviors.

### The main effect of the reference groups of descriptive norms: Closer-range advantage

It is essential to specify the reference group for the effectiveness of descriptive norms, and we found that compared with distal group norms, individuals influenced by proximal group norms had stronger public health compliance behaviors, which is similar to the findings of established studies (Yun and Silk, 2011; Zhuang, 2022). In the pandemic context, individuals pay more attention to the behaviors of proximal group members who are physically closer to them. Their attention is focused on the information related to the proximal group, so that proximal group norms might directly and deeply affect individual decision-making. In addition, individuals and proximal group members share similar public health policies and preventive and control measures, which can make individual behavior more deeply influenced by the behavior of proximal groups. Therefore, when individuals perceive that most people around them are actively following public health policies, individuals may also show stronger public health compliance behaviors.

### The interaction effect of the properties and reference groups of descriptive norms: Self-protection first

In addition to the main effects of the property and the reference group on individual behaviors, this study also revealed the interaction between the two on public health compliance behaviors, and specifically, that proximal group norms can diminish the difference in the effect of positive and negative norms on compliance behaviors. In the context of repeated impact of epidemics and the occasional viral outbreaks, public health behaviors are closely related to individual life and health (Kowalski and Black, 2021); noncompliance behaviors of proximal groups pose a significant health threat to individuals, and at this time individuals can only take more proactive and strict compliance measures to effectively mitigate health threats. The findings of the interaction imply that protection motivation can override social motivation for behaviors that are closely related to life and health. Although existing studies have indicated that descriptive norms tend to unconsciously preferentially guide individual behaviors and be more effective than injunctive norms (Mollen et al., 2013; Lac and Donaldson, 2018), the influence of descriptive norms is often closely tied to the specific context of the behavior. In a context closely related to life and health, the priority role of descriptive norms may need to be re-examined when descriptive norms contradict injunctive norms. In this study, the condition of negative-proximal group norms can be seen as a conflict between descriptive norms that most people around do not comply with public policy and injunctive norms that individuals should actively adopt public health compliance behaviors. When the proximal group generally adopts noncompliance behaviors, the individuals’ strong self-protection motivation plays a dominant role to maintain their health, and at this time, individuals tend to violate descriptive norms under social motivation and prefer to comply with the injunctive norms under protection motivation. According to the theory of planned behavior, motivation is an important factor influencing the effectiveness of social norms (Ajzen, 1991). Therefore, when individuals perceive the health-adverse behaviors from the proximal group, they have a weak motivation to comply with others’ behaviors, and instead are more willing to adopt protective behaviors. At this time, the difference in the effect of positive and negative norms on public health compliance behaviors is diminished. The finding of this study for the interaction implies that the role of descriptive norms in a public health context should be viewed with caution when they are inconsistent with injunctive norms.

### The mediating role of risk perception: Stimulation of threat

Moreover, we revealed the mechanism by which descriptive norms affect public health compliance behaviors from the perspective of risk perception, validating the applicability of protection motivation theory in public health (Rogers, 1983). The significant mediating role of risk perception explains the incentive effect of the negative norm on individual compliance behaviors and illustrates that for behaviors related to life and health, the individual’s protection motivation dominantly influences the individual’s compliance. The prevalence of the majority’s noncompliance with public health policies could activate their perception of risks in the environment, so as to adopt strict health behaviors to protect themselves. Furthermore, proximal group norms could reinforce the role of negative norms on risk perception. When individuals find that others who share close physical space with them are not undertaking protective measures as required by public health policy, they might feel that the threat is more serious and closer to them, and their susceptibility may be further strengthened, leading to greater recommended risk-reducing behaviors. Additionally, we did not find a significant mediating role of risk perception in the association between the reference group and public health compliance behaviors in this study. We assume that the possible reason is that epidemic diseases spread quickly and widely, and when diseases spread within a city, its residents tend to bind together as a community with a shared future and adopt the same response, so behaviors of other citizens in this city can also influence individual perceptions of the disease and judgments of riskiness. Similar to the current COVID-19 pandemic, once a few cases of infection appear in a city, the government would take strict control measures targeting a large number of residents, such as prohibiting random movement and requiring nucleic acid testing (Yu and Li, 2020). At this time, citizens would be highly sensitive to the disease and reach a high level of risk perception, even though the infected people may be far away from them. Thus, the role of norms across different physical distance reference groups on risk perception may not be significantly different, in which case the mediation model is not significant.

### The moderating effect of behavioral visibility: Secretly dismiss the norm

This study also indicated that for high-visibility behaviors, individual compliance would be higher, and the effect of the property of descriptive norms on compliance behaviors would be stronger. The results of behavioral visibility provide more empirical research support for previous studies’ findings on behavioral visibility (Wismans et al., 2020), and imply the importance of increasing visibility to promote public health compliance behavior. An individual’s compliance with public health behaviors is heavily influenced by whether they can be observed by others. When a behavior is highly visible, individuals are more willing to show compliance to conform to social expectations, while when compliance is difficult to observe, individuals may develop slackness and fail to comply with public health behaviors. Moreover, information on descriptive norms creates social pressure on the individual’s decision-making environment (Blais et al., 2018; Ahmad Rizal et al., 2022). When a behavior is visible and most people are actively complying with it, the individual compliance pressure rapidly increases, which is conducive to compliance behaviors. However, if it is difficult for others to observe whether the individual is performing the behavior, the pressure to conform may be reduced and individual compliance behavior may be weakened. Furthermore, this study did not support the moderating effect of behavioral visibility on the association between the reference group of descriptive norms and public health compliance behaviors. We think that the possible reason is that hand washing or hand sanitizing is always emphasized and supervised in places like homes or organizations where individuals spend time with the proximal group (Guo and Zhang, 2022) so that hand hygiene behaviors which were originally low in visibility become easily observable in the proximal group. Therefore, the role of proximal group norms might do not diminish in the condition of hygiene behaviors. Thus, for high-visibility and low-visibility behaviors, the facilitative effect of proximal group norms might always be stronger than the effect of distal group norms, so the moderating role of behavioral visibility in the effect of the reference group of descriptive norms might be not significant.

## Theoretical contribution and implications

Based on a behavioral public policy perspective, we attempt to use descriptive norms to achieve implicit guidance of public health compliance behaviors, and specifically find that both the property and the reference group of descriptive norms can significantly influence individual compliance behaviors. Furthermore, we reveal that risk perception is a key mechanism for descriptive norms to function, and that behavioral visibility is an important applicable condition of descriptive norms. Both in theory and application, this study is an expansion of existing studies on descriptive norms and public compliance.

From a theoretical perspective, we refine the study of social norms and explore the effectiveness of descriptive norms in nudging public health compliance behaviors in terms of their properties and reference groups, which provides a new grounding for the application of behavioral public policy in the field of public health. Moreover, we integrate the focus theory of normative conduct and the protection motivation theory to construct and examine a new model of public health compliance behaviors, which reveals the mechanisms and conditions of compliance behaviors in the public health context. Aristotle in his Politics says this: “Man is by nature a social animal.” This study, to a certain degree, responds to the classic philosophical proposition of the relationship between “sociality” and “self-interest.” Sociality is an important attribute of humans, whose behavioral decisions are influenced by social groups and tend to conform to the behaviors of the majority. However, human is still an animal in nature with the attribute of seeking benefits and avoiding harm. In an uncertain and risky environment, self-interest and self-protection motivation may both play the most central role.

According to our results, we suggest that for public health compliance behaviors, descriptive norms are a potentially effective nudge strategy. In general, when governments want to motivate individuals to participate in public health behaviors, they can simply highlight the prevalence of the behavior and create positive normative environments in which most people are actively complying with public health policies, thus driving individual compliance. In addition, the construction of normative information should focus on proximal groups that are physically closer to individuals and guide them to pay attention to the compliance behaviors of those around them. Meanwhile, when publicizing public health policies, the government should reach out to places with a more focused scope, such as residential communities and office buildings, to improve the precision of publicity. However, it is worth noting that when individuals perceive widespread noncompliance from proximal groups, their risk perception is extremely strong, and this insecurity from the surrounding environment dominates public health compliance behaviors. This suggests that the authorities should focus on the important role of individual risk perception in promoting the implementation of public health policies and stimulate individual self-protection motivation by exposing the proximal group’s noncompliance behaviors. However, the use of risk perception should be moderate to avoid unnecessary panic. Moreover, the characteristics of public health behavior itself can also affect public health compliance behaviors, and increasing the visibility of public health behaviors can effectively promote individual compliance. Therefore, the use of descriptive norms should take into account the visibility of public health behaviors: even though information on descriptive norms creates social pressure for individuals to decide whether to comply with public health behaviors, the effectiveness of descriptive norms will be greatly reduced if the behavior itself is not easily perceived and observed by others. Therefore, the government can devote itself to improving the visibility of key public health behaviors, for example, by prominently displaying hand hygiene disinfectants in public places and publicizing public health behavior data, to maximize the effect of descriptive norms on individual compliance behavior.

Providing information on descriptive norms and increasing the visibility of public health behaviors are common nudging strategies: the former uses a cross-sectional comparison of social norm frames, and the latter draws on changes in the physical environment (Lehner et al., 2016; Gao and Wang, 2021). Although our study reconfirms the effectiveness of both strategies in nudging public health compliance behaviors, it cannot ignore the potential ethical risks involved. For instance, positive norms may run the risk of ignoring withdrawal, and negative norms may lead to intentional intimidation. However, increasing transparency in the use of social norms, that is, disclosing the way social norms work and the purpose of using them, might inhibit the nudging effect (Kantorowicz-Reznichenko and Kantorowicz, 2021). Therefore, there is a trade-off in the use of nudging strategies, and under the premise of proper ethical guidelines, they can be an effective policy tool to achieve public policy goals (Fu et al., 2022). In the field of public health, these ethical guidelines need to strike a balance between respecting individual autonomy, preventing health risks, and promoting health obligations (Baum et al., 2007; Jaffe and Hope, 2010; Hansen et al., 2016).

## Limitations and future studies

We acknowledge some limitations to this study. First and foremost, the method of this study was manipulating experimental variables in a virtual situation. Although all experimental materials passed the manipulation check, we could not determine whether subjects processed these materials differently from their responses to actual intervention information. In the future, field experiments should be considered to allow subjects to receive actual materials in realistic situations to examine the consistency of the research findings. Besides, in addition to risk perception, other mechanisms might link descriptive norms to public health compliance. For example, other cognitive factors such as normative beliefs may be a significant mediator (Nolan, 2011) and may even play a chain mediating role with risk perception. Additionally, descriptive norms may change individual behaviors by influencing individuals’ emotions (Poškus et al., 2019), and cognitive and emotional factors may form a dual-mediation model. Future studies could refer to these research ideas and include more variables to further analyze the mechanisms of descriptive norms. In addition, future research could consider other moderators to more fully explore the applicable conditions under which the descriptive specification functions. For example, the effect of descriptive norms on individual behaviors might not only be moderated by physical distance, but variables related to an individual’s psychological distance from the group, such as social identity, may also play a significant moderating role (Liu et al., 2019). Variables focusing on the characteristics of the behavior itself, such as its acceptability, may also be moderators worth considering (Rimal, 2008). Moreover, in cross-cultural research contexts, researchers can discuss whether there is a significant difference in the role of descriptive norms in societies with different cultural values (Wang et al., 2022).

## Data availability statement

The raw data supporting the conclusions of this article will be made available by the authors, without undue reservation.

## Ethics statement

The studies involving human participants were reviewed and approved by Institutional Review Board Office, School of Government, Sun Yat-sen University. The patients/participants provided their written informed consent to participate in this study.

## Author contributions

All authors have made a substantial, direct, and intellectual contribution to the work, and approved the manuscript for publication.

## Funding

This study was supported by the National Natural Science Foundation of China (Grant No. 71873149) and the National Social Science Fund of China (Grant No. 18CGL043).

## Conflict of interest

The authors declare that the research was conducted in the absence of any commercial or financial relationships that could be construed as potential conflicts of interest.

## Publisher’s note

All claims expressed in this article are solely those of the authors and do not necessarily represent those of their affiliated organizations, or those of the publisher, the editors and the reviewers. Any product that may be evaluated in this article, or claim that may be made by its manufacturer, is not guaranteed or endorsed by the publisher.

## Supplementary material

The Supplementary material for this article can be found online at: https://www.frontiersin.org/articles/10.3389/fpsyg.2022.1040218/full#supplementary-material
