# Supplementary material for: Follow or not? Descriptive norms and public health compliance: Mediating role of risk perception and moderating effect of behavioral visibility
Source: Front Psychol. 2022 Nov 18;13:1040218. doi: 10.3389/fpsyg.2022.1040218 (PMC9717382; doi:10.3389/fpsyg.2022.1040218)
Supplement: Supplementary file 1 [file Data_Sheet_1.pdf]

Appendix 1. Complete experimental materials.

The introductory paragraph and picture for experimental conditions involving the low-visibility behavior:

You are a citizen of City A. The recent spring influenza virus outbreak in City A has resulted in some cases of community and family and friend transmission. The government has decided to strengthen the city's hand hygiene management to prevent and control influenza. All public places in City A have placed hand sanitizers in accordance with the government requirements of hand hygiene standards, and the government has also posted reminders and popular science posters on hand hygiene on bulletin boards. Citizens are also asked to strengthen their personal hand hygiene management.

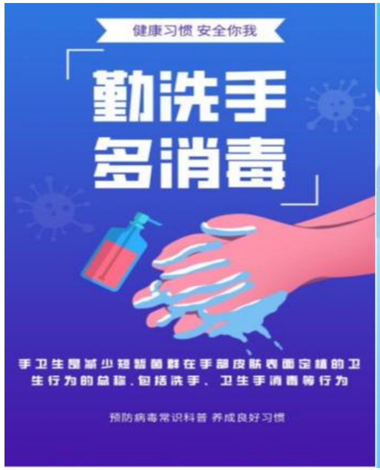

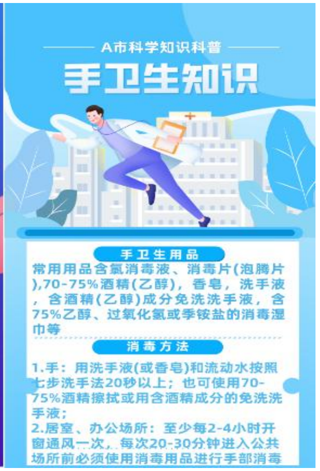

The experimental material 1 (negative norm × distal group × low visibility):

You read a news report published in the morning newspaper about a survey on citizens' hand hygiene management in City A. The data indicates that over 75% of the citizens do not pay much attention to hand hygiene management and do not comply with strict hand hygiene measures. Most people do not use free hand sanitizer when they go to public places because they are in a hurry, lack attention, or do not think it is necessary. The news also simulcast interviews with randomly selected citizens yesterday about whether they are concerned about their hand hygiene. Most interviewed citizens said: "I do not pay much attention to hand hygiene, and I usually only wash my hands before and after meals and toilets. I do not have a disinfectant hand sanitizer at home, and I just rinse my hands with water." Other interviewees said that they seldom notice hand sanitizers in public places, and even if they see them, most of the time they just ignore them and do not use them. They do not pay much attention to whether they touch their eyes, nose, and mouth with their hands in public places. Similar to survey data, most citizens are not

concerned about the health effects of hand hygiene and do not strictly manage their own. You also observed that the disinfection products in major supermarkets in City A are unsalable.

The experimental material 2 (positive norm × distal group × low visibility):

You read a news report published in the morning newspaper about a survey on citizens' hand hygiene management in City A. The data indicates that over 75% of the citizens pay close attention to hand hygiene management and actively comply with strict hand hygiene measures, especially when going into public places. The news also simulcast interviews with randomly selected citizens yesterday about whether they are concerned about their hand hygiene. Most interviewed citizens said: "I pay great attention to hand hygiene, and the first thing I do when I get home from work is to wash my hands carefully with sanitizer. I also stock a lot of sanitizing hand sanitizer at home, and I strictly follow the seven-step hand-washing method for more than 20 seconds when I have enough time." Other interviewees said that in public places they use hand sanitizer every time to disinfect their hands. They try to avoid touching their eyes, nose, and mouth with their hands in public places. Similar to survey data, most citizens are concerned about the health effects of hand hygiene and strictly manage their own. You also observed that the disinfection products in major supermarkets in City A are salable.

The experimental material 3 (negative norm × proximal group × low visibility):

You read a community notification issued by the community committee about a survey on residents' hand hygiene management in your community. The data indicates that over 75% of the residents do not pay much attention to hand hygiene management and do not comply with strict hand hygiene measures. Most residents do not use free hand sanitizer when they go into community spaces because they are in a hurry, lack attention, or do not think it is necessary. The community committee also sent out videos of the residents being interviewed in the WeChat group. The neighbor who lives above you said: "I do not pay much attention to hand hygiene, and I usually only wash my hands before and after meals and toilets. I do not have a disinfectant hand sanitizer at home, and I just rinse my hands with water." Other neighbors who live in the next building said that they seldom notice hand sanitizers in the community, and even if they see them, most of the time they just ignore them and do not use them. They do not pay much attention to whether they touch their eyes, nose, and mouth with their hands in the community. Similar to survey data, most residents are not concerned about the health effects of hand hygiene and do not strictly manage their own. You also observed that the disinfection products in convenience stores in your community are unsalable. Meanwhile, you observed in the office and at family and friend gatherings that most of your colleagues, friends, and family members do not carry hand hygiene products with them.

The experimental material 4 (positive norm × proximal group × low visibility):

You read a community notification issued by the community committee about a survey on residents' hand hygiene management in your community. The data indicates that over 75% of the residents pay close attention to hand hygiene management and actively comply with strict

hand hygiene measures, especially when going into community spaces. The community committee also sent out videos of the residents being interviewed in the WeChat group. The neighbor who lives above you said: “I pay great attention to hand hygiene, and the first thing I do when I get home from work is to wash my hands carefully with sanitizer. I also stock a lot of sanitizing hand sanitizer at home, and I strictly follow the seven-step hand-washing method for more than 20 seconds when I have enough time.” Other neighbors who live in the next building said that in the community they use hand sanitizer every time to disinfect their hands. They try to avoid touching their eyes, nose, and mouth with their hands in the community. Similar to survey data, most residents are concerned about the health effects of hand hygiene and strictly manage their own. You also observed that the disinfection products in convenience stores in your community are salable. Meanwhile, you observed in the office and at family and friend gatherings that most of your colleagues, friends, and family members carry hand hygiene products with them.

The introductory paragraph and picture for experimental conditions involving the high-visibility behavior:

You are a citizen of City A. The recent spring influenza virus outbreak in City A has resulted in some cases of community and family and friend transmission. The government has decided to strengthen the city’s mask-wearing management to prevent and control influenza. The government insists that wearing a mask can effectively prevent the spread of the virus and reduce cold air inhalation, without adverse influence on physical activities. City A has repeatedly reported the spread of diseases due to the irregular wearing of masks, which has triggered a heated debate. The government has also posted reminders and popular science posters on mask-wearing on bulletin boards. Citizens are also asked to strengthen their personal mask-wearing management.

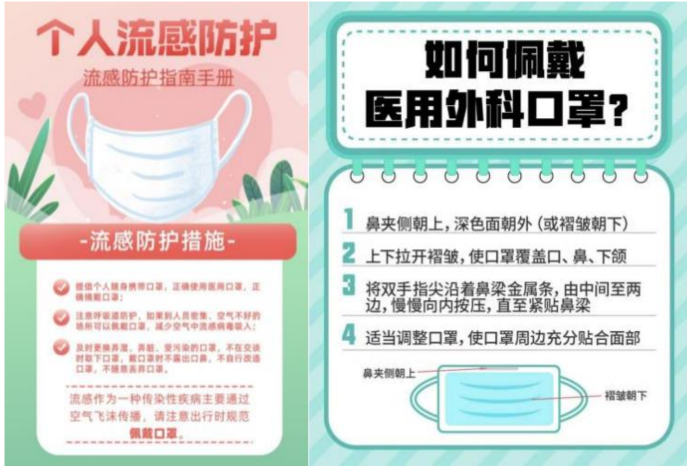

The experimental material 5 (negative norm × distal group × high visibility):

You read a news report published in the morning newspaper about a survey on citizens' mask-wearing management in City A. The data indicates that over 75% of the citizens do not pay much attention to mask-wearing management and do not comply with strict mask-wearing measures. The news also simulcast interviews with randomly selected citizens yesterday about whether they are concerned about wearing a mask. Most interviewed citizens said: "I often forget to take a mask when going to public places, and if I take it, I just put it in my bag." Other interviewees said that because wearing masks is uncomfortable, they often wear a mask at the checkpoints of public places, but take it off when they enter, and most of the citizens they saw were not wearing a mask in public places. Similar to survey data, the majority (32 citizens) of the 40 citizens photographed in a random photo in a major supermarket in City A are not wearing a mask, and the probability is higher in outdoor places such as streets. You also observed that medical masks in major pharmacies in City A are unsalable.

The experimental material 6 (positive norm × distal group × high visibility):

You read a news report published in the morning newspaper about a survey on citizens' mask-wearing management in City A. The data indicates that over 75% of the citizens pay close attention to mask-wearing management and actively comply with strict mask-wearing measures, especially when going into public places. The news also simulcast interviews with randomly selected citizens yesterday about whether they are concerned about wearing a mask. Most interviewed citizens said: "I pay great attention to mask-wearing. I always take a few masks when going to public places, and I often remind others to wear a mask." Other interviewees said that they wear compliant medical masks everywhere except at home or in open areas where masks are not necessary, and most of the citizens they saw were wearing a mask in public places. Similar to survey data, the majority (32 citizens) of the 40 citizens photographed in a random photo on the street in City A are wearing a mask, and the probability is higher in indoor places such as major supermarkets. You also observed that medical masks in major pharmacies in City A are salable.

The experimental material 7 (negative norm × proximal group × high visibility):

You read a community notification issued by the community committee about a survey on residents' mask-wearing management in your community. The data indicates that over 75% of the residents do not pay much attention to mask-wearing management and do not comply with strict mask-wearing measures. The community committee also sent out videos of the residents being interviewed in the WeChat group. The neighbor who lives above you said: "I often forget to take a mask in the community, and if I take it, I just put it in my bag." Other neighbors who live in the next building said that because wearing masks is uncomfortable, they often wear a mask at the checkpoint at the community gate, but take it off when they enter, and most of the residents they saw were not wearing a mask in the community. Similar to survey data, the majority (32 residents) of the 40 residents photographed in a random photo in an elevator in your community are not wearing a mask. You observed that medical masks in community pharmacies are unsalable. Meanwhile, you observed in the office and at family and friend gatherings that most of

your colleagues, friends, and family members do not wear masks at all times and do not remove masks properly.
**The experimental material 8 (positive norm × proximal group × high visibility):**<br>You read a community notification issued by the community committee about a survey on residents' mask-wearing management in your community. The data indicates that over 75% of the residents pay close attention to mask-wearing management and actively comply with strict mask-wearing measures, especially when going into community spaces. The community committee also sent out videos of the residents being interviewed in the WeChat group. The neighbor who lives above you said: "I pay great attention to mask-wearing. I always take a few masks when going into the community, and I often remind others to wear a mask." Other neighbors who live in the next building said that they wear compliant medical masks everywhere except at home or in open areas where masks are not necessary, and most of the residents they saw were wearing a mask in the community. Similar to survey data, the majority (32 residents) of the 40 residents photographed in a random photo in an elevator in your community are wearing a mask. You observed that medical masks in community pharmacies are salable. Meanwhile, you observed in the office and at family and friend gatherings that most of your colleagues, friends, and family members wear masks at all times and remove masks properly.

Appendix 2. Measurement for the main variables.

Variable	Item (As a resident of City A, …)
Manipulation check[a]	I think [most people in this city] / [most people among my neighbors, friends, family members, and colleagues] follow [hand hygiene behaviors] / [mask-wearing behaviors].<br>I think [the citizens in this city] / [neighbors, friends, family members, and colleagues] are physically close to me.<br>I think others can notice whether [I am wearing a mask] / [I am practicing hand hygiene].
Public health compliance behaviors (hand hygiene)[b]	I will clean my hands regularly and thoroughly with an alcohol-based hand sanitizer.<br>I will avoid touching my eyes, nose, and mouth with unwashed hands.<br>I will cover my mouth and nose with tissues or my bent elbow when coughing or sneezing instead of using my hands.<br>I will often use hand cleaning products and wash my hands for at least 20 seconds.<br>In the past week, I washed my hands frequently.
Public health compliance behaviors (mask-wearing)[b]	I will wear a mask at social gatherings.<br>I will wear a mask when I go to public places even during non-peak hours.<br>I will wear a mask in crowded and poorly ventilated spaces.<br>If I am at risk of getting sick, I will wear a mask.<br>In the past week, I wore a mask when I went out.
Risk perception[b]	I am concerned that others do not comply with [hand hygiene behaviors] / [mask-wearing behaviors].<br>I think I am at risk of contracting a disease within the next one month from not [practicing hand hygiene] / [wearing a mask].<br>I think my friends and family members are at risk of contracting a disease within the next one month from not [practicing hand hygiene] / [wearing a mask].<br>I don't think it would affect many people even if I do not [practice hand hygiene] / [wear a mask].<br>I could contract a disease by not [practicing hand hygiene] / [wearing a mask].<br>It is serious for me to contract a disease.

[a]Items were rated on a 7-point scale ranging from 1 (*strongly disagree*) to 7 (*strongly agree*).

[b]Items were rated on an 11-point scale ranging from 1 (*strongly disagree*) to 11 (*strongly agree*).
